# Development and implementation of a digiphysical screening model with nationwide reach to diagnose familial hypercholesterolemia

**DOI:** 10.1177/20552076241311156

**Published:** 2025-01-23

**Authors:** Karin Littmann, Gustav Kindborg, Matthias Lidin, Linda Mellbin, Daniel Eriksson Hogling, Jonas Brinck

**Affiliations:** 1 Department of Medicine Huddinge, 27106 Karolinska Institutet, Stockholm, Sweden; 2 Medical Unit of Endocrinology, 59562 Karolinska University Hospital, Stockholm, Sweden; 3 Department of Cardiology, 59562 Karolinska University Hospital, Stockholm, Sweden; 4 Department of Medicine Solna, Karolinska Institutet, Stockholm, Sweden

**Keywords:** Cardiovascular disease, digital health, screening, familial hypercholesterolemia, digiphysical screening model

## Abstract

**Background:**

Familial hypercholesterolemia (FH) is a hereditary dyslipidemia that confers a severely elevated risk for development of early atherosclerotic cardiovascular disease if left untreated. FH is underdiagnosed in most countries including Sweden.

**Aim:**

To develop and evaluate the implementation of a digiphysical screening model to diagnose FH in the clinical routine.

**Methods:**

A digiphysical screening model for FH, containing digital and physical related activities was developed and fully implemented in routine clinical care in the Stockholm region, Sweden 2022. The centerpiece of the model is a tailormade interactive web-based platform designed to facilitate communication and secure medical information exchange between its participants and the healthcare professionals. The screening model includes, (i) cascade screening of relatives to patients with a confirmed FH diagnosis and (ii) systematic selective screening of patients with established atherosclerotic coronary artery disease.

**Results:**

Until October 2023, 338 index patients were included in the cascade screening. They invited 954 relatives nationwide, 616 (64.6%) accepted participation, 346 (36.3%) were completely screened, and 141 (14.8%) have received a FH diagnosis (40.8% of all completely screened). Selective screening was performed in 2867 patients with coronary artery disease, 355 (12.4%) were identified with increased risk for FH and underwent a genetic test. Of these, 153 (3.8%) had a genetic test result and 52 (1.8%) were diagnosed with FH.

**Conclusions:**

A digiphysical screening model with a nationwide reach to diagnose FH was successfully implemented in routine clinical care. The model has potential to facilitate FH screening and provide health economic benefits long term.

## Introduction

Familial hypercholesterolemia (FH) is a hereditary dyslipidemia characterized by elevated plasma cholesterol levels from birth.^1^ The trait induces development of premature atherosclerotic cardiovascular disease with predisposition for coronary artery disease (CAD) if left untreated.^2,3^ Early diagnosis and initiation of cholesterol lowering treatment can alter the natural course of the disease and prevent development of atherosclerosis.^4^ The gold standard for diagnosing autosomal dominant FH is genetic testing^5^ but the most common method used worldwide is still scoring-based algorithms for a clinical FH diagnosis.^6^ However, a negative genetic test does not exclude the FH diagnosis which means that all individuals undergoing investigation needs to be individually assessed by a doctor.

## Background

The FH disease is severely underdiagnosed in most parts of the world despite its huge negative impact on cardiovascular health.^7^ With an estimated prevalence for heterozygous FH of 1 in 311 in the general population and 1 in 16 in patients with established atherosclerotic CAD, the challenges with FH screening can be perceived as overwhelming.^8^ In Sweden in 2023, FH was estimated to be diagnosed in less than 20% of the predicted national population.^9^ Adaptive care models have been described to address the global challenge to diagnose FH^10^ and digital technologies have been commended to play an increasing role in this endeavor.^11^ Considering this, we developed a systematic digiphysical screening model with nationwide reach to diagnose FH in the clinical routine. We present the structure of the model, the results from the first 2 years of screening and discuss future perspectives (Clinicaltrials.gov NCT04929457).

## Methods

### Development and description of the digiphysical screening model

**Figure 1.**
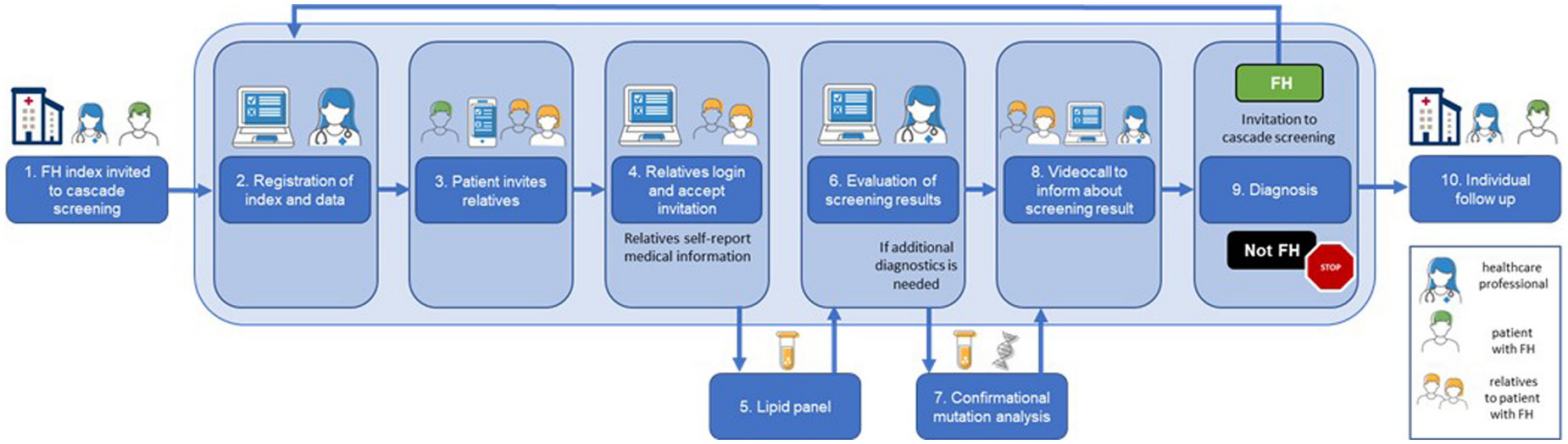
Overview of the digiphysical cascade screening model of relatives to patients with familial hypercholesterolemia (FH). The light-blue rectangle denotes activities performed digitally on the interactive web-based platform or by phone/video call whereas physical meetings or activities are visualized outside the rectangle. (1) A patient with FH is informed and invited to participate in screening at a routine visit for medical care by becoming an index. (2) The index's personal and medical information is registered via the healthcare provider's interface on the web-based platform. (3) The index accesses the patient’s interface of the web-based platform, creates a family tree and invite relatives to participate in the screening. The relatives receive a digital invitation by text message or email. (4) The invited relative accesses the relative’s interface of the web-based platform, accepts participation in screening and self-reports medical information about any cardiovascular family history, cardiometabolic disease and any lipid lowering treatment. Thereafter the relative receives a referral for blood sampling for a lipid panel. (5) The relative leaves a blood sample at a local laboratory and test results are automatically transferred to the healthcare provider's interface of the web-based platform. (6) A doctor evaluates screening results and decides if the diagnosis of FH can be confirmed or acquitted based on the available information or if a confirmational genetic FH test is needed. (7) A confirmational genetic test s is performed. (8) The relative is informed about the screening results and diagnosis of FH, or acquittal, on the platform and at a videocall. (9) Relatives diagnosed with FH are invited to become a new index in the cascade screening. If acquitted of FH, the screening stops. (10) An individual plan for treatment and followup is established.

**Figure 2.**
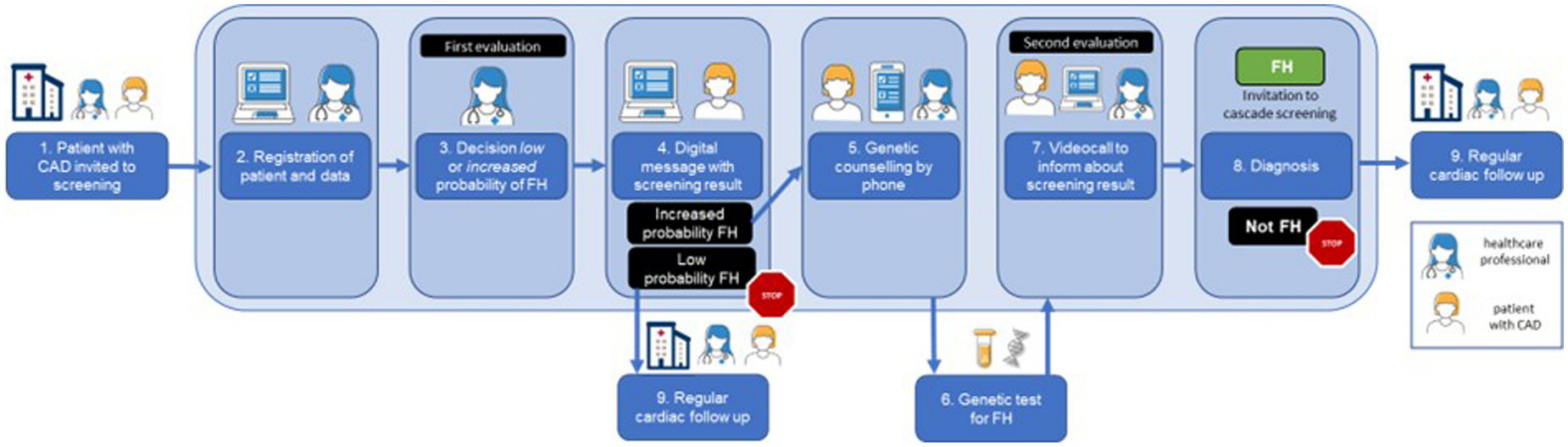
Overview of the digiphysical selective screening model for familial hypercholesterolemia (FH) of patients with atherosclerotic coronary artery disease (CAD). The light-blue rectangle denotes activities performed digitally on the interactive web-based platform or by phone/video call whereas physical meetings or activities are visualized outside the rectangle. (1) A patients with CAD is informed and invited to participate in screening at a routine visit for cardiac care. (2) The patient's personal and medical information is registered via the healthcare provider's interface on the web-based platform. (3) A doctor performs a first evaluation of the probability for the patient to have FH (low or increased probability). (4) The patient receives a digital message by text or email to access screening result via the patient's interface of the web-based platform. If low probability for FH, the screening ends. (5) Patients with increased probability for FH are given genetic counseling by phone and recommended a genetic test. (6) The patient leaves a blood sample for genetic test at a local laboratory which forwarded to the centralized laboratory for analysis. (7) A doctor performs a second and definite evaluation and sets an FH diagnosis or acquittal and the patient received the information during a videocall. (8) Patients diagnosed with FH are invited to become an index in the cascade screening. (9) All patients, regardless of FH status, continue with their routine cardiac followup.

The digiphysical FH screening model includes (i) cascade screening of relatives to index patients with a confirmed FH diagnosis (Figure 1) and (ii) systematic selective screening of patients with established atherosclerotic CAD (Figure 2). The digiphysical screening model was developed as an innovation project at Karolinska University Hospital, Stockholm, Sweden and then gradually implemented in clinical routine care in the Stockholm region in Sweden. In this manuscript we present the digiphysical screening model including data from cascade screening of relatives to index patients with a confirmed FH diagnosis from August 2021 and data from systematic screening of patients with established atherosclerotic CAD from April 2022 until October 2023. The centerpiece of the model is a tailormade CE-marked (Conformité Européenne) clinical decision tool designed as an interactive web-based platform (www.FHabian.nu) that facilitate efficient communication and internet secure information exchange between the healthcare professional and the participants in the screening. The web-based platform is accessed via any web-browser and comprises an interface for its participants undergoing screening, and an interface for the healthcare professional. It contains clinical data (described below) needed for a doctor to fully evaluate participants medically and to confirm or acquit the FH diagnosis.

### Legal and ethical aspects of the digiphysical screening model and internet security

A thorough legality review was carried out by a lawyer during the development of the digiphysical screening model. The General Data Protection Regulation (GDPR)^12^ and the Swedish Patient Data Act were considered. The Data Protection Officer is a representative at Karolinska University Hospital. All individuals give written consent to participation in the digiphysical screening program after been given oral and/or written information before registration on the web-based platform. The participants deposit and access data on the platform with a personal e-identification (eID) that requires a Swedish personal identification number. The model also allows legal guardians to give consent to participation of their under-aged children to participate in screening. The written consent is obtained digitally through the interactive web-based platform. Individuals who decline consent are not included and no data on them are stored. Ethical approval to perform research on clinical data collected in the digiphysical screening program is given from the Swedish Ethical Review Authority (no. 20230022601). The healthcare professional accesses the platform by similar e-authentication used for the standard electronical medical records in Sweden. All data on the platform is securely stored on data servers (SjuNet^®^) in Sweden that also store electronic medical records used by all Swedish healthcare Regions.

### Information exchange in the digiphysical screening model

The interactive web-based platform is the centerpiece of the digiphysical screening model and forms the foundation for data deposit and communication between all its participants and the healthcare professional. Index patients with a confirmed FH diagnosis in the cascade screening (Figure 1) are registered in the web-based platform by the healthcare professional and can then invite relatives on the web-based platform to participate in screening. The invited relatives can, by clicking the invitation link received by text messages for phone or email, accept participation. An automatically generated reminder is sent after 2 weeks if the invitation is unrequited. First after acceptance, the healthcare professional can access information about the relatives. Individuals who refrain from login are not considered participants of screening and the healthcare professional will have no information about them. Patients with CAD participating in selective screening (Figure 2) are registered on the web-based platform by a healthcare professional in conjunction with a routine clinical visit for cardiac care.

### Inclusion criteria for participation in the digiphysical screening

- Informed consent: for children aged <13 years the legal guardians give informed consent.
- A Swedish personal identity number and a personal eID. Children <18 years without a personal eID can be administrated by the legal guardian's eID.
- In cascade screening: a confirmed diagnosis of FH for the index, and biological relationship to the index for the invited relative.
- In the systematic selective screening: established atherosclerotic CAD and being a habitant in the Stockholm region in Sweden.

### Exclusion criteria for participation in the digiphysical screening

- Unable to understand the Swedish language.
- No access to eID and internet connection.

### Medical information collection, blood sampling, laboratory analyses, and genetic testing

For index patients with an FH diagnosis and for patients with CAD, medical information is registered on the web-based platform by a healthcare professional based on their medical records. It includes lifestyle factors, smoking status, cardiovascular comorbidities, and any cholesterol lowering therapy. For relatives participating in cascade screening, the medical information is self-reported.

The plasma lipids reported for index patients and patients with CAD are collected from their medical records while relatives participating in cascade screening have plasma lipids analyzed *de novo*. Relatives receive a digital referral or a referral by postal mail depending on residential area in Sweden for blood sampling to measure plasma lipids (total cholesterol, low-density lipoprotein -cholesterol (LDL), high-density lipoprotein-cholesterol (HDL), triglycerides, and lipoprotein(a)). Blood sampling is performed at any local routine clinical laboratory nationwide and all laboratory results are digitally transferred directly into the web-based platform. If no laboratory test results are received within 3 months a reminder is sent to the relatives by postal mail. Participants who are recommended for a genetic test are contacted by phone by a healthcare professional and receive genetic counseling. The referral is sent by postal mail, the blood sampling is performed at any local laboratory. The blood is sent in a padded envelope to Translational Analysis in Molecular Medicine (TAMM), Karolinska University Hospital, Stockholm, Sweden. The genetic test for FH is a two-step analysis with a mutation analysis panel, including 119 known mutations and deletions in the three most important FH genes: the LDL-receptor (LDLR;NM_000527.4), apolipoprotein B (APOB;NM_000384.3), and the Proprotein convertase subtilisin/kexin type 9 (PCSK9;NM_174936.3) performed with the Agena MassARRAY system and iPLEX Gold chemistry (MassARRAYAgena Bioscience, San Diego, California, USA). If no mutation is detected in the panel analysis, the sample is analyzed with the Devyser's FH v2 amplicon sequencing methodology using Illumina's MiSeq instrument. The assay specifically detects substitutions and INDELS in exons and intron–exon boundaries of the genes for LDLR (exon 1–18), PCSK9 (exon 1–12), and APOB (exon 26–29). If the index patient has a known mutation, a mutation confirmation test for the mutation is performed in the relatives.^13^

### Assessments and documentation of screening results

All participants evaluated for FH in the digiphysical screening are reviewed by a licenced doctor that diagnose FH or acquit based on available information on the platform. The evaluation process involves several steps where a genetic test for FH can be added at the discretion of the doctor (Figures 1 and 2). All assessments are communicated to participants digitally via the web-based platform. If the diagnosis of FH is confirmed, a video or physical visit with a doctor at the Endocrinology Unit, Karolinska University Hospital, Stockholm, Sweden is organized. The relative is informed about the diagnosis, and depending on where the relative live in Sweden, referred to the local specialist center for treatment. Information generated in the screening activities and saved on the web-based platform is documented in the participants’ electronical medical record in parallel.

### Data management and statistics

Data for this manuscript was managed in GraphPad Prism ver. 9.0 (Dotmatics, GraphPad Software, 225 Franklin Street. Fl. 26, Boston, MA 02110) and Microsoft Excel (Microsoft 365, Windows). Descriptive data from the first 2 years of screening are presented as number (n) and percent.

## Results

**Figure 3.**
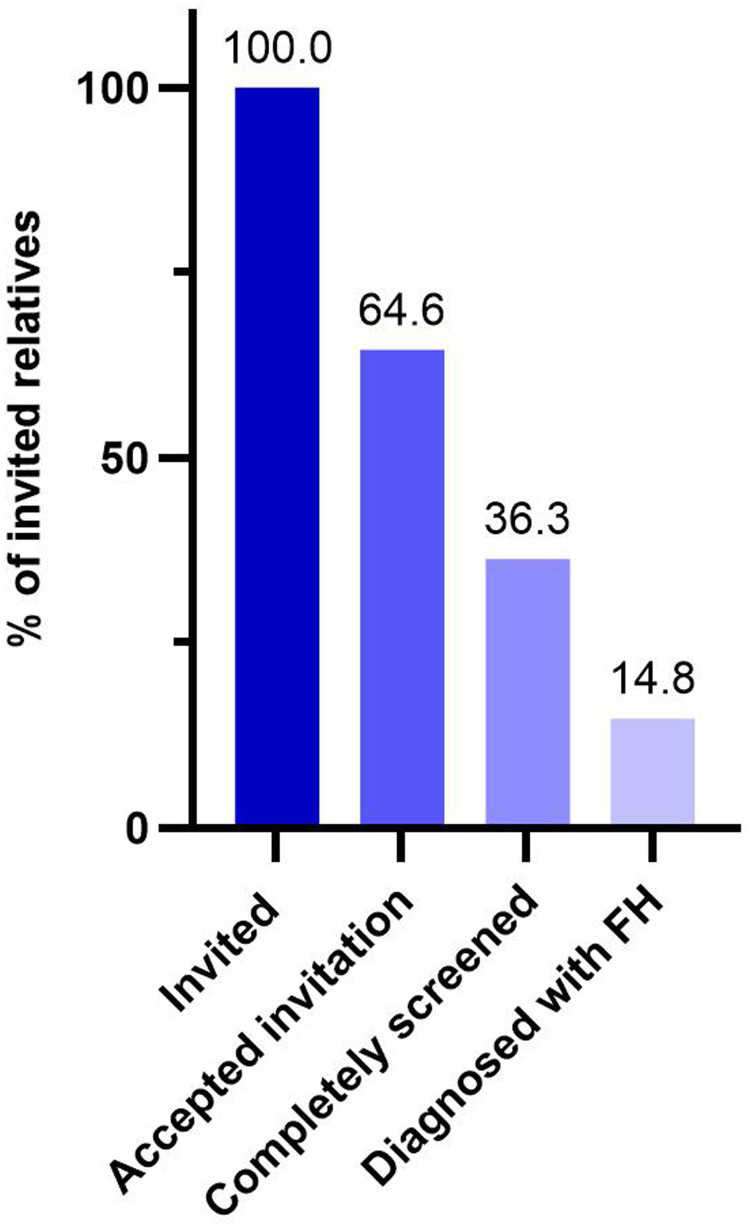
Participation and diagnostic yield of familial hypercholesterolemia (FH) in digiphysical cascade screening of relatives. Bars show proportion of all relatives invited to screening by the index patient (n=954), those who accepted invitation (n=616), those who were completely screened (n=346), and the relatives diagnosed with FH (n=141).

Digiphysical screening for FH was fully introduced into clinical routine care at six hospitals with cardiac care in April 2022 in Region Stockholm. Two years after introduction of the cascade screening program (Figure 1), 338 index patients with a confirmed FH diagnosis (mean age 50.4 years, males 44.4%) have been included. From the 954 invited relatives, 616 (64.6%) accepted the invitation (mean age 34.3 years, males 46.6%), and the screening are completed in 346 (36.3%) (mean age 36.5, males 44.2%). In total 141 relatives (mean age 38.2 years, males 20.6%) have been diagnosed with FH (40.8% of completely screened, 22.9% of those accepted invitation, and 14.8% of all invited) (Figure 3).

**Figure 4.**
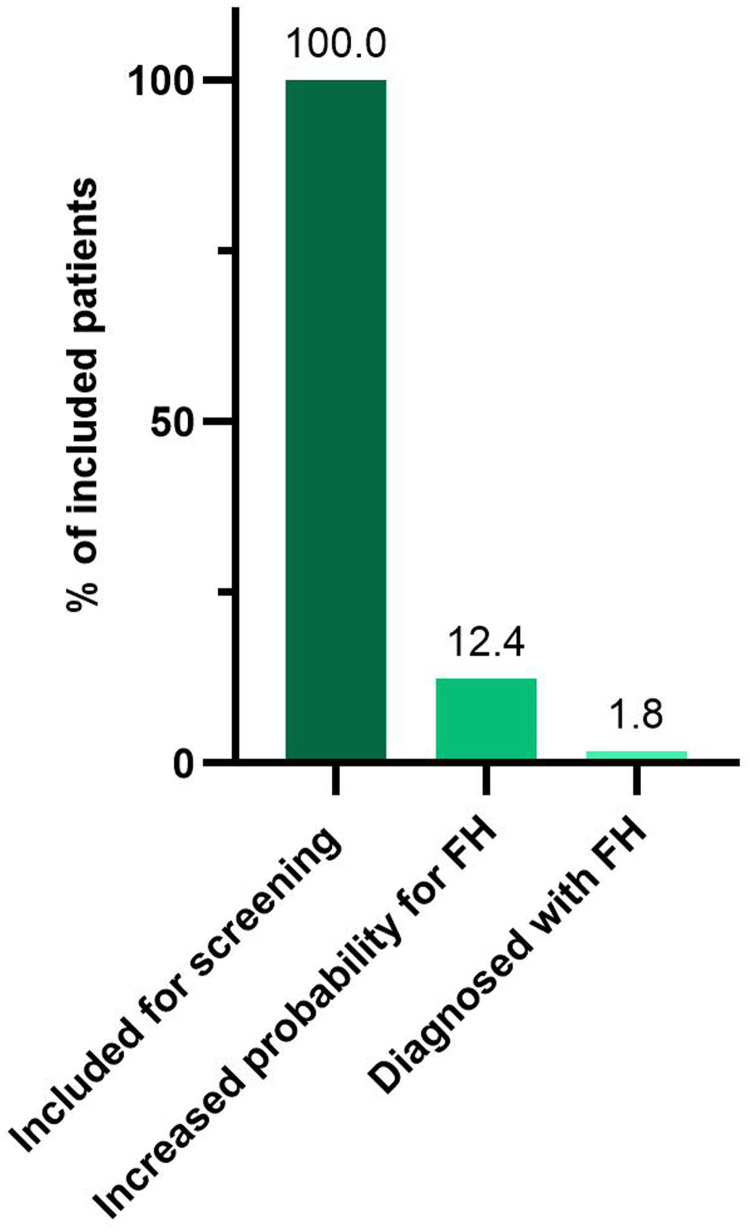
Participation and diagnostic yield of familial hypercholesterolemia (FH) in selective screening of patients with coronary artery disease. Bars show proportion all included patients by a healthcare professional (n=2867), those evaluated to have a low (n=2512) or increased (n=355) probability for FH, and the patients diagnosed with FH (n=52).

In the systematic selective screening program (Figure 2), 2867 patients with CAD have been included (mean age 65.1 years, males 79.2%). In the first assessment, 2512 (87.6%, mean age 65.9 years, males 81.4%) were categorized to have low probability and 355 (12.4%. mean age 58.1, males 58.3%) to have increased probability for FH. For 153 (43.1%) patients with increased probability for FH, a completed genetic test result was available, 52 (34.0%, mean age 58.9 years, males 69.2%) have been diagnosed with FH and in 101 (66.0%, mean age 58.7 years, males 72.3%) the diagnosis has been acquitted (Figure 4). Genetic test results are awaiting in 202 (56.9%) patients with increased probability for FH. If the same proportion of FH diagnosis, that is, 34.0% of all individuals with increased probability for FH and available genetic test result, is assumed in those with increased probability awaiting genetic test results as those with available genetic test result, a total of 121 patients (34.0% of 355) would be diagnosed with FH. It corresponds to 4.2% of all patients included for selective screening.

## Discussion

Our digiphysical screening model displays several advantages. The tailormade interactive web-based platform enhances the access to screening, digitalizes and automatizes key activities of screening, and facilitates communication of medical information between its participants and the healthcare provider. The platform also aggregates all accumulated screening data and presents it to the healthcare professional who assesses the participants which allows for standardized evaluations with limited personnel resources. Finally, the model allows for a single center to coordinate and to perform cascade screening with a nationwide reach by including relatives on a national basis. The use of digital portals in patient care have been suggested to be favorable.^14^

The legal and privacy aspects of the screening activities performed in the present digiphysical screening model have been addressed and clarified before their implementation in the clinical routine. It includes the consideration of General Data Protection Regulation (GDPR) and the responsibilities of the Data Protection Officer. The digiphysical model also circumvents the prohibition for a healthcare provider to directly contact and invite relatives to cascade screening that exists in many countries^15–17^ by letting index patients invite their own relatives which resolves this issue. Integrity concerns could hypothetically be raised whether family members are psychologically troubled to participate in screening or if an FH diagnosis impairs the quality of life but this has not been the case in previously studies.^18,19^ On the contrary, family members have been reported to be positive to participation in FH screening.^17^

The participation of relatives in digiphysical cascade screening was approximately 65% in this study which a lower than what has been reported from the national FH screening project in the Netherlands (85%).^20^ However, the screening in the Netherlands was based on nondigital activities by the healthcare provider including telephone calls and home-visits which is labor intense. The digiphysical cascade screening model is labor minimalistic due to its digitalization and the patient-mediated approach of inviting relatives but still provides comparable participation as seen in direct-contact cascade screening programs.^21^ More intense work-up on nonparticipants including repeated automatically generated reminders and personal contacts might potentially increase participation rates, a matter for future development.

Regional context, timing and funding may influence the feasibility and implementation of an innovation project, like the digiphysical screening model described herein, into the clinical routine care. Several nationwide and regional actions have been made to increase the awareness of FH in Sweden. Since 2015 the Swedish National Board of Health and Welfare have been given FH screening a high priority in the national guidelines for clinical cardiology.^22^ The Swedish patient organization for FH work actively to increase the national awareness of this and have participated in the dialogue with regional politicians facilitating the implementation of the digiphysical screening model into routine clinical care in the Stockholm region. In addition, national and regional media have drawn attention to the digiphysical screening model, and it has been presented in national and international scientific contexts.^9,23^ In the Stockholm region, six hospitals treating patients with established atherosclerotic CAD are involved in the screening program and regional guidelines for screening and treatment of FH are implemented. Taken together, these factors may have influenced the willingness to participate in the digiphysical screening model from both patients and healthcare providers perspectives. A limitation of the current digiphysical screening model is that participation requires basic knowledge in the Swedish language, a Swedish personal identification number and access to an electronical identification (eID), which excludes a small proportion of the population.

## Conclusion

In conclusion, a digiphysical screening model with nationwide reach to diagnose FH was successfully developed and implemented in the clinical routine and shows valid screening outcomes in its initial phase. We expect to include >6000 participants during 3 years of screening after which the digiphysical screening model will be fully evaluated focusing on screening efficacy, cost-effectiveness, and health economics. The inclusiveness, structure, and systematic approach of the model will allow for population-based results and generalizable conclusions.

## References

[1] Nordestgaard BG Chapman MJ Humphries SE , et al. Familial hypercholesterolaemia is underdiagnosed and undertreated in the general population: guidance for clinicians to prevent coronary heart disease: consensus statement of the European Atherosclerosis Society. Eur Heart J 2013; 34: 3478–390a. 23956253 10.1093/eurheartj/eht273 PMC3844152

[2] Krogh HW Mundal L Holven KB , et al. Patients with familial hypercholesterolaemia are characterized by presence of cardiovascular disease at the time of death. Eur Heart J 2016; 37: 1398–1405. 26586781 10.1093/eurheartj/ehv602

[3] Collaboration EASFHS . Global perspective of familial hypercholesterolaemia: a cross-sectional study from the EAS Familial Hypercholesterolaemia Studies Collaboration (FHSC). Lancet 2021; 398: 1713–1725. 34506743 10.1016/S0140-6736(21)01122-3

[4] Luirink IK Wiegman A Kusters DM , et al. 20-YearfFollow-up of statins in children with familial hypercholesterolemia. N Engl J Med 2019; 381: 1547–1556. 31618540 10.1056/NEJMoa1816454

[5] Abifadel M Boileau C . Genetic and molecular architecture of familial hypercholesterolemia. J Int Med 2023; 293: 144–165. 10.1111/joim.13577 PMC10092380 36196022

[6] Khera AV Hegele RA . What is familial hypercholesterolemia, and why does it matter? Circulation 2020; 141: 1760–1763. 32479201 10.1161/CIRCULATIONAHA.120.046961 PMC7299543

[7] Representatives of the Global Familial Hypercholesterolemia C Wilemon KA Patel J , et al. Reducing the clinical and public health burden of familial hypercholesterolemia: a global call to action. JAMA Cardiol 2020; 5: 217–229. 31895433 10.1001/jamacardio.2019.5173

[8] Hu P Dharmayat KI Stevens CAT , et al. Prevalence of familial hypercholesterolemia among the general population and patients with atherosclerotic cardiovascular disease: a systematic review and meta-analysis. Circulation 2020; 141: 1742–1759. 32468833 10.1161/CIRCULATIONAHA.119.044795

[9] Littmann K Kindborg G Nathanson D , et al. Digiphysical cascade screening model to diagnose familial hypercholesterolemia in relatives to an index case. Lakartidningen 2022; 119: 21210. 35471725

[10] Watts GF Gidding SS Mata P , et al. Familial hypercholesterolaemia: evolving knowledge for designing adaptive models of care. Nat Rev Cardiol 2020; 17: 360–377. 31974482 10.1038/s41569-019-0325-8

[11] Ibrahim S Reeskamp LF Stroes ESG , et al. Advances, gaps and opportunities in the detection of familial hypercholesterolemia: overview of current and future screening and detection methods. Curr Opin Lipidol 2020; 31: 347–355. 33027222 10.1097/MOL.0000000000000714

[12] Brauneck A Schmalhorst L Kazemi Majdabadi MM , et al. Federated machine learning, privacy-enhancing technologies, and data protection laws in medical research: scoping review. J Med Internet Res 2023; 25: e41588. 10.2196/41588 PMC10131784 36995759

[13] Benedek P Eriksson M Duvefelt K , et al. Genetic testing for familial hypercholesterolemia among survivors of acute coronary syndrome. J Intern Med 2018; 284: 674–684. 29974534 10.1111/joim.12812

[14] Carini E Villani L Pezzullo AM , et al. The impact of digital patient portals on health outcomes, system efficiency, and patient attitudes: updated systematic literature review. J Med Internet Res 2021; 23: e26189. 10.2196/26189 PMC8459217 34494966

[15] Leren TP Bogsrud MP . Cascade screening for familial hypercholesterolemia should be organized at a national level. Curr Opin Lipidol 2022; 33: 231–236. 35942821 10.1097/MOL.0000000000000832

[16] Leren TP Bogsrud MP . The importance of cascade genetic screening for diagnosing autosomal dominant hypercholesterolemia: results from twenty years of a national screening program in Norway. J Clin Lipidol 2021; 15: 674–681. 34479846 10.1016/j.jacl.2021.08.007

[17] Louter L Defesche J Roeters van Lennep J . Cascade screening for familial hypercholesterolemia: practical consequences. Atheroscler Suppl 2017; 30: 77–85. 29096865 10.1016/j.atherosclerosissup.2017.05.019

[18] van Maarle MC Stouthard ME Bonsel GJ . Quality of life in a family based genetic cascade screening programme for familial hypercholesterolaemia: a longitudinal study among participants. J Med Genet 2003; 40: e3. 10.1136/jmg.40.1.e3 PMC1735254 12525551

[19] van Maarle MC Stouthard ME Marang-van de Mheen PJ , et al. How disturbing is it to be approached for a genetic cascade screening programme for familial hypercholesterolaemia? Psychological impact and screenees’ views. Community Genet 2001; 4: 244–252. 12751487 10.1159/000064200

[20] Umans-Eckenhausen MA Defesche JC Sijbrands EJ , et al. Review of first 5 years of screening for familial hypercholesterolaemia in the Netherlands. Lancet 2001; 357: 165–168. 11213091 10.1016/S0140-6736(00)03587-X

[21] Frey MK Ahsan MD Bergeron H , et al. Cascade testing for hereditary cancer syndromes: should we move toward direct relative contact? A systematic review and meta-analysis. J Clin Oncol 2022; 40: 4129–4143. 35960887 10.1200/JCO.22.00303 PMC9746789

[22] *Welfare SNBoHa* . Nationella riktlinjerför hjärtsjukvård www.socialstyrelsen.se Swedish National Board of Health and Welfare 2018.

[23] Kindborg G. Abstract: development and implementation of a digiphysical screening model with nationwide reach to diagnose familial hypercholesterolemia. Atherosclerosis 2024; 395 117627. 10.1177/20552076241311156 PMC11758524 39866892

